# Associations Between Dementia, Race-Ethnicity, and Intensive and Patient-Centered End-of-Life Care

**DOI:** 10.1093/geroni/igab046.1273

**Published:** 2021-12-17

**Authors:** Elizabeth Luth, Amanda Reich, Robert Semco, Holly Prigerson, Joel Weissman, Adoma Manful

**Affiliations:** 1 Rutgers University, New Brunswick, New Jersey, United States; 2 Harvard Medical School/Brigham and Women’s Hospital, Boston, Massachusetts, United States; 3 Weill Cornell Medicine, New York, New York, United States

## Abstract

A retrospective cohort analysis of Medicare administrative claims data from 2016-2018 compared intensive and patient-centered end-of-life care measures in persons with and without dementia, including the moderating effects of race/ethnicity. Over half (53%) of 485,209 Medicare decedents had a dementia diagnosis. Decedents with dementia were 31-34% less likely to receive intensive end-of-life care (hospital death 95%CI: 0.64-0.67; hospitalization in last 30 days 95%CI: 0.68-0.70) and 50% more likely to receive timely hospice care (95%CI: 1.48-1.52). The association between dementia and end-of-life care varied by decedent race/ethnicity. Compared to non-Hispanic white decedents without dementia, non-Hispanic Black, Hispanic and Asian decedents with dementia were significantly more likely to receive intensive end-of-life care. Non-Hispanic Black decedents with dementia were 23% more likely to receive timely hospice care (95%CI: 1.11-1.36). Additional research is needed to understand why persons with dementia receive less intensive end-of-life care and why differences exist based on racial/ethnic status.

